# Theoretical requirements and inverse design for broadband perfect absorption of low-frequency waterborne sound by ultrathin metasurface

**DOI:** 10.1038/s41598-018-37510-w

**Published:** 2019-02-04

**Authors:** Jie Zhong, Honggang Zhao, Haibin Yang, Yang Wang, Jianfei Yin, Jihong Wen

**Affiliations:** Vibration and Acoustics Research Group, Laboratory of Science and Technology on Integrated Logistics Support, National University of Defense Technology, Changsha, 410073 China

## Abstract

Effective absorption of low-frequency waterborne sound with subwavelength absorbers has always been a challenging work. In this paper, we derive two theoretical requirements for broadband perfect absorption of low-frequency waterborne sound by ultrathin acoustic metasurface under a finite-thickness steel plate followed by semi-infinite air. Based on the theoretical requirements, an acoustic metasurface, a rubber layer embedded periodically with cavities, is inversely designed to achieve perfect absorption at 500 Hz. The metasurface is as thin as 1% of the working wavelength and maintains a substantially high absorptance over a relatively broad bandwidth. The perfect absorption peak is attributed to the overall resonance mode of the metasurface/steel plate system. Besides, high absorption can still be achieved even if the loss factor of the given rubber material cannot meet the ideal requirement. Finally, a strategy to utilize the inherent frequency-dependent characteristics of dynamic parameters of rubber material is suggested to achieve an ultra-broadband perfect absorption. When the frequency-dependent characteristics of the given rubber matrix cannot meet the theoretical requirements, a broadband super-absorption can still be realized by properly designing the frequency position of perfect absorption of the cavity-based metasurface.

## Introduction

Effective absorption of low frequency noise with a subwavelength absorber has always been a challenge, owing to the difficulty in achieving impedance matching and the inherently weak intrinsic dissipation of linear materials at low frequency domain^1,2^. Conventional means of acoustic absorption use porous materials^3^, gradient index materials^4,5^, or micro-perforated panels^6^. Such absorbers usually result in bulky dimensions comparable with the incoming wavelength. For more than one decade, the rapid expansion of acoustic metamaterials and metasurfaces has paved a way to manipulate the acoustic wave in unprecedented ways such as negative refraction^7–9^, subwavelength imaging^10,11^, cloaking^12,13^, and one-way transmission^14,15^. Metasurfaces are generally thin structures having subwavelength thickness consisting of unit cells that could give rise to numerous intriguing phenomena^16,17^. With an emphasis on their planarity and ultrathin thickness, metasurfaces are also claimed by some researchers as the planarized version of metamaterials, because both exhibit unusual properties^18,19^. One important category of acoustic metasurfaces is the absorptive metasurface^17,19–45^, which exhibits super absorption for incident waves within a deep subwavelength thickness. Over the past few years, a host of absorptive metasurfaces have been proposed such as membrane-type resonators^20–23^, coiled-up Helmholtz resonators and Fabry-Pérot channels^17,24–32^, coherent perfect absorbers^33,34^ and metaporous materials^35–37^.

Currently, most absorptive acoustic metasurfaces are designed for airborne sound, very few acoustic metasurfaces are aimed at efficiently absorbing low-frequency sound in water, in which the working wavelength is always larger than that in air at a same frequency, and the fluid-structure interaction becomes non-negligible. With an emphasis on subwavelength property, a bubble metascreen, consisting of soft elastic layer periodically embedded with air bubbles, was theoretically and experimentally verified to exhibit super-absorption in waterborne ultrasound^45^. Very recently, using an inverse design strategy (from performances to structures), Mei *et al*. proposed a new design of highly absorptive metasurface for waterborne sound possessing a thickness 0.15 of the working wavelength and a 21% relative bandwidth for over 80% of absorption^19^.

It is noteworthy that the abovementioned two research works are both based on an ideal reflective (hard wall) boundary. Nevertheless, for the case the metasurfaces are bonded to the hull of an underwater vehicle, the hull vibration should not be neglected when the analysis is performed in low-frequency regime. Actually, it has been found by several researchers that the vibration of the steel plate of finite thickness has a significant effect on the acoustic absorptance of the rubbery coatings in low frequency range^46–50^. Therefore, it is more reasonable to treat the metasurface and the hull as a whole when the low-frequency acoustic performances are investigated. Though this effect has already been reported elsewhere, to the best of the authors’ knowledge, no one has already utilized the effect of the vibration of steel plate for inverse design of absorptive acoustic metasurface.

Based on the aforementioned considerations, this paper focuses on the perfect absorption of low-frequency waterborne sound (below 1500 Hz) within a deep subwavelength thickness. Firstly, we derive the theoretical requirements for broadband perfect absorption of waterborne sound by ultrathin acoustic metasurface under a finite-thickness steel plate followed by semi-infinite air. Then, based on the theoretical requirements, an acoustic metasurface, consisting of a rubber layer embedded periodically with cavities, is inversely designed to achieve perfect absorption at 500 Hz. Different from the iterative search used in ref.^19^, the inverse design method proposed in this paper is based on the formulas of theoretical requirements, thus no further iterative search is needed. The mechanism for perfect absorption and the effect of non-ideal loss factor on sound absorption are also investigated. Finally, a strategy, utilizing the inherent frequency-dependent characteristics of dynamic parameters of rubber material, is suggested to achieve an ultra-broadband perfect absorption.

## Theoretical Requirements for Perfect Absorption

**Figure 1:**
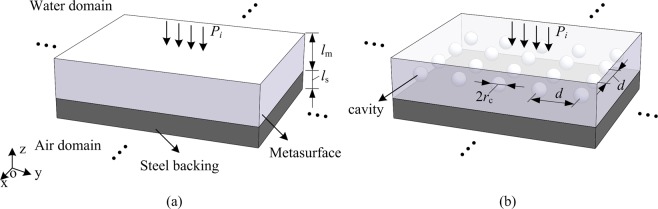
(**a**) A schematic view of the underwater absorptive metasurface with a finite-thickness steel plate followed by air. (**b**) A physical realization of the proposed metasurface for perfect absorption.

Figure 1(a) shows the schematic description of the problem. An absorptive acoustic metasurface is water-loaded at one side and bonded to a finite-thickness steel plate followed by semi-infinite air. The structure considered is infinite in *xoy* plane. The thicknesses of the metasurface and the steel plate are *l*_m_ and *l*_*s*_ respectively. A harmonic plane wave is normally incident from the semi-infinite water domain.

Due to the characteristic impedance of air is much less than that of the steel and *k*_s_*l*_s_ ≪ 1 at low frequencies, the input impedance of the finite-thickness steel plate for normal incidence is^47,49^

1 $${Z}_{{\rm{in}}}^{{\rm{s}}}={\rm{j}}{\rho }_{{\rm{s}}}{c}_{{\rm{s}}}\,\tan ({k}_{{\rm{s}}}{l}_{{\rm{s}}})\approx {\rm{j}}{\rho }_{{\rm{s}}}\omega {l}_{{\rm{s}}},$$

where *ρ*_s_, *c*_s_ and *k*_s_ are respectively the mass density, longitudinal wave speed and longitudinal wavenumber of steel, ${\rm{j}}=\sqrt{-1}$ is the imaginary unit, *ω* is the angular frequency.

The corresponding input impedance on the metasurface, according to the well-known impedance transfer formula, is given by

2 $${Z}_{{\rm{in}}}={\rho }_{{\rm{m}}}{c}_{{\rm{m}}}\frac{{Z}_{{\rm{in}}}^{{\rm{s}}}+{\rm{j}}{\rho }_{{\rm{m}}}{c}_{{\rm{m}}}\,\tan ({k}_{{\rm{m}}}{l}_{{\rm{m}}})}{{\rho }_{{\rm{m}}}{c}_{{\rm{m}}}+{\rm{j}}{Z}_{{\rm{in}}}^{{\rm{s}}}\,\tan ({k}_{{\rm{m}}}{l}_{{\rm{m}}})}={\rho }_{{\rm{m}}}{c}_{{\rm{m}}}\frac{{\rm{j}}{\rho }_{{\rm{s}}}\omega {l}_{{\rm{s}}}+{\rm{j}}{\rho }_{{\rm{m}}}{c}_{{\rm{m}}}\,\tan ({k}_{{\rm{m}}}{l}_{{\rm{m}}})}{{\rho }_{{\rm{m}}}{c}_{{\rm{m}}}-({\rho }_{{\rm{s}}}\omega {l}_{{\rm{s}}})\,\tan ({k}_{{\rm{m}}}{l}_{{\rm{m}}})},$$

in which *ρ*_m_, *c*_m_ and *k*_m_ are respectively the mass density, longitudinal wave speed and longitudinal wavenumber in metasurface. Considering the low-frequency waterborne sound application and the deep subwavelength of metasurface, i.e. |*k*_m_*l*_m_| ≪ 1, the equation (2) can be simplified as

3 $${Z}_{{\rm{in}}}\approx {\rho }_{{\rm{m}}}{\tilde{c}}_{{\rm{m}}}^{{\rm{2}}}\frac{{\rm{j}}({\rho }_{{\rm{s}}}{l}_{{\rm{s}}}+{\rho }_{{\rm{m}}}{l}_{{\rm{m}}})\omega }{{\rho }_{{\rm{m}}}{\tilde{c}}_{{\rm{m}}}^{{\rm{2}}}-{\rho }_{{\rm{s}}}{\omega }^{{\rm{2}}}{l}_{{\rm{s}}}{l}_{{\rm{m}}}}=\frac{{\rho }_{{\rm{m}}}{c}_{{\rm{m}}}^{{\rm{2}}}({\rho }_{{\rm{s}}}{l}_{{\rm{s}}}+{\rho }_{{\rm{m}}}{l}_{{\rm{m}}})\omega (\,-\,{\eta }_{{\rm{m}}}+{\rm{j}})}{{\rho }_{{\rm{m}}}{c}_{{\rm{m}}}^{{\rm{2}}}(1+{\rm{j}}{\eta }_{{\rm{m}}})-{\rho }_{{\rm{s}}}{\omega }^{{\rm{2}}}{l}_{{\rm{s}}}{l}_{{\rm{m}}}}.$$

The complex wave speed ${\tilde{c}}_{{\rm{m}}}={c}_{{\rm{m}}}\sqrt{1+{\rm{j}}{\eta }_{{\rm{m}}}}$, with *η*_m_ standing for the loss factor of the longitudinal modulus of the metasurface, is used here to characterize the damping in the metasurface.

For a plane wave normally incident onto the metasurface, the pressure reflection coefficient R can be evaluated as

4 $$R=\frac{{Z}_{{\rm{in}}}-{Z}_{{\rm{w}}}}{{Z}_{{\rm{in}}}+{Z}_{{\rm{w}}}},$$

where *Z*_w_ = *ρ*_w_*c*_w_ is the specific acoustic impedance of water, *ρ*_w_ and *c*_w_ are respectively the mass density and wave speed in water. Inserting equation (3) into equation (4), one can further obtain

5 $$R=\frac{{M}_{{\rm{m}}}({\rho }_{{\rm{m}}}{l}_{{\rm{m}}}+{\rho }_{{\rm{s}}}{l}_{{\rm{s}}}){\eta }_{{\rm{m}}}\omega +{Z}_{{\rm{w}}}{M}_{{\rm{m}}}-{Z}_{{\rm{w}}}{l}_{{\rm{s}}}{l}_{{\rm{m}}}{\rho }_{{\rm{s}}}{\omega }^{2}+[{Z}_{{\rm{w}}}{M}_{{\rm{m}}}{\eta }_{{\rm{m}}}-{M}_{{\rm{m}}}({\rho }_{{\rm{m}}}{l}_{{\rm{m}}}+{\rho }_{{\rm{s}}}{l}_{{\rm{s}}})\omega ]{\rm{j}}}{{M}_{{\rm{m}}}({\rho }_{{\rm{m}}}{l}_{{\rm{m}}}+{\rho }_{{\rm{s}}}{l}_{{\rm{s}}}){\eta }_{{\rm{m}}}\omega -{Z}_{{\rm{w}}}{M}_{{\rm{m}}}+{Z}_{{\rm{w}}}{l}_{{\rm{s}}}{l}_{{\rm{m}}}{\rho }_{{\rm{s}}}{\omega }^{2}-[{Z}_{{\rm{w}}}{M}_{{\rm{m}}}{\eta }_{{\rm{m}}}+{M}_{{\rm{m}}}({\rho }_{{\rm{m}}}{l}_{{\rm{m}}}+{\rho }_{{\rm{s}}}{l}_{{\rm{s}}})\omega ]{\rm{j}}},$$

with ${M}_{m}={\rho }_{m}{c}_{m}^{2}$ standing for the real part of the longitudinal modulus (or the longitudinal dynamic modulus) of the metasurface.

Note that the transmission coefficient can be ignored due to the large impedance mismatch between the steel plate and air, the sound absorption coefficient can be calculated by *A* ≈ 1 − |*R*|^2^, which means that a perfect absorption can be obtained when reflection acquiring zero. Thus, from the condition *R* = 0, two equations can be separately derived by putting the real and imaginary part of the numerator in the right part of equation (5) to be zero

6 $${M}_{{\rm{m}}}({\rho }_{{\rm{m}}}{l}_{{\rm{m}}}+{\rho }_{{\rm{s}}}{l}_{{\rm{s}}}){\eta }_{{\rm{m}}}\omega +{Z}_{{\rm{w}}}{M}_{{\rm{m}}}-{Z}_{{\rm{w}}}{l}_{{\rm{s}}}{l}_{{\rm{m}}}{\rho }_{{\rm{s}}}{\omega }^{{\rm{2}}}=0,$$

7 $${Z}_{{\rm{w}}}{M}_{{\rm{m}}}{\eta }_{{\rm{m}}}-{M}_{{\rm{m}}}({\rho }_{{\rm{m}}}{l}_{{\rm{m}}}+{\rho }_{{\rm{s}}}{l}_{{\rm{s}}})\omega =0.$$

For a metasurface with the given thickness *l*_m_, the theoretical requirements (*M*_m_ and *η*_m_) of the metasurface for broadband perfect absorption can be derived from the equations (6) and (7),

8 $${M}_{{\rm{m}}}^{{\rm{p}}}=\frac{{Z}_{{\rm{w}}}{l}_{{\rm{s}}}{l}_{{\rm{m}}}{\rho }_{{\rm{s}}}{\omega }^{2}}{{Z}_{{\rm{w}}}+({\rho }_{{\rm{m}}}{l}_{{\rm{m}}}+{\rho }_{{\rm{s}}}{l}_{{\rm{s}}})\omega {\eta }_{{\rm{m}}}},$$

9 $${\eta }_{{\rm{m}}}^{{\rm{p}}}=\frac{({\rho }_{{\rm{m}}}{l}_{{\rm{m}}}+{\rho }_{{\rm{s}}}{l}_{{\rm{s}}})\omega }{{Z}_{{\rm{w}}}},$$

with superior letter “p” standing for the requirement of perfect absorption. Inserting Eq (9) into (8), a decoupled condition for ${M}_{{\rm{m}}}^{{\rm{p}}}$ can be deduced as,

10 $${M}_{{\rm{m}}}^{{\rm{p}}}=\frac{{Z}_{{\rm{w}}}^{{\rm{2}}}{\rho }_{{\rm{s}}}{l}_{{\rm{s}}}{l}_{{\rm{m}}}{\omega }^{{\rm{2}}}}{{Z}_{{\rm{w}}}^{{\rm{2}}}+{({\rho }_{{\rm{m}}}{l}_{{\rm{m}}}+{\rho }_{{\rm{s}}}{l}_{{\rm{s}}})}^{{\rm{2}}}{\omega }^{{\rm{2}}}}.$$

For a given thickness *l*_m_, the equations (9) and (10) thus prescribe the frequency-dependent relations of the material parameters of the metasurface (*ρ*_m_, ${M}_{{\rm{m}}}^{{\rm{p}}}$ and ${\eta }_{{\rm{m}}}^{{\rm{p}}}$). When these two conditions can be simultaneously satisfied in a broad range of frequencies, the broadband perfect absorption can thus be achieved.

## Results

### Inverse design for perfect absorption at a single frequency

The above derivation leads to the frequency-dependent conditions prescribed by equations (9) and (10), for broadband perfect absorption. However, how to realize such specific parameters, especially the frequency-dependent relations, remains an unresolved issue. The following statements will show that the viscoelastic rubber materials, considering their good impedance match with water and inherent frequency-dependent characteristic of material parameters, combined with properly embedded spherical cavities can be a promising choice for the broadband perfect absorption under a steel plate. Figure 1(b) shows the schematic view of the proposed structures, where a rubber matrix is periodically embedded with air cavities. The radius and the period for square arrangement of the cavity are *r*_c_ and *d*.

In the long-wavelength limit, the effective mass density *ρ*_e_, effective bulk modulus ${\tilde{K}}_{{\rm{e}}}={K}_{{\rm{e}}}(1+{\rm{j}}{\eta }_{{\rm{K}}}^{{\rm{e}}})$ and effective shear modulus ${\tilde{\mu }}_{{\rm{e}}}={\mu }_{{\rm{e}}}(1+{\rm{j}}{\eta }_{{\rm{\mu }}}^{{\rm{e}}})$, with *K*_e_ and *μ*_e_ denoting the effective storage moduli, ${\eta }_{{\rm{K}}}^{{\rm{e}}}$ and ${\eta }_{{\rm{\mu }}}^{{\rm{e}}}$ denoting the effective loss factors, of the rubber layer periodically embedded with spherical air cavities can be derived using the static effective formula from ref.^51^ as

11 $${\rho }_{{\rm{e}}}=\varphi {\rho }_{0}+(1-\varphi )\rho \approx (1-\varphi )\rho ,$$

12 $${\tilde{K}}_{{\rm{e}}}=\tilde{K}+\frac{\varphi ({K}_{0}-\tilde{K})}{1+3(1-\varphi )({K}_{0}-\tilde{K})/(3\tilde{K}+4\tilde{\mu })}\approx \frac{4\tilde{K}\tilde{\mu }(1-\varphi )}{3\varphi \tilde{K}+4\tilde{\mu }},$$

13 $${\tilde{\mu }}_{{\rm{e}}}=\tilde{\mu }+\frac{5\varphi \tilde{\mu }({\mu }_{0}-\tilde{\mu })}{5\tilde{\mu }+6(1-\varphi )({\mu }_{0}-\tilde{\mu })(\tilde{K}+2\tilde{\mu })/(3\tilde{K}+4\tilde{\mu })}\approx \frac{(9\tilde{K}+8\tilde{\mu })\tilde{\mu }(1-\varphi )}{(9\tilde{K}+8\tilde{\mu })+6\varphi (\tilde{K}+2\tilde{\mu })},$$

where, *ρ*_0_, *K*_0_ and *μ*_0_ are respectively the mass density, bulk modulus and shear modulus of the air; *ρ*, $\tilde{K}=K(1+{\rm{j}}{\eta }_{{\rm{K}}})$ and $\tilde{\mu }=\mu (1+{\rm{j}}{\eta }_{{\rm{\mu }}})$ are respectively the mass density, complex bulk modulus and complex shear modulus of the viscoelastic rubber with *K* and *μ* denoting the storage moduli, *η*_K_ and *η*_μ_ denoting the loss factors; $\varphi =\tfrac{4}{3}\pi {r}_{{\rm{c}}}^{{\rm{3}}}/({l}_{{\rm{m}}}{d}^{2})$ is the volume fraction of air cavity. Here, *ρ*_0_, *K*_0_ and *μ*_0_ of the air are assumed to be zero for simplicity.

The effective longitudinal modulus can be obtained using the relation

14 $${\tilde{M}}_{{\rm{e}}}={\tilde{K}}_{{\rm{e}}}+4{\tilde{\mu }}_{{\rm{e}}}/3.$$

If one rewrites ${\tilde{M}}_{{\rm{e}}}$ into the form ${\tilde{M}}_{{\rm{e}}}={M}_{{\rm{e}}}(1+{\rm{j}}{\eta }_{{\rm{M}}}^{{\rm{e}}})$, then *M*_e_ and ${\eta }_{{\rm{M}}}^{{\rm{e}}}$ denotes the effective longitudinal dynamic modulus and loss factor, respectively. Considering the rubber material with an isotropic loss factor, i.e., *η*_K_ = *η*_μ_ = *η*^52,53^, one can easily get the real-number relations, using equations (12)~(14), as

15 $${M}_{{\rm{e}}}={K}_{{\rm{e}}}+\frac{4}{3}{\mu }_{{\rm{e}}}=\frac{4K\mu (1-\varphi )}{3\varphi K+4\mu }+\frac{4}{3}\frac{(9K+8\mu )\mu (1-\varphi )}{(9K+8\mu )+6\varphi (K+2\mu )},$$

16 $${\eta }_{{\rm{M}}}^{{\rm{e}}}=\eta .$$

From equations (11)~(14), one can find the effective material parameters of the inhomogeneous viscoelastic rubber layer vary with *ϕ*. Thus, for a given viscoelastic rubber material, one can adjust its effective material parameters by varying the volume fraction *ϕ* to meet the theoretical requirements of perfect absorption, i.e., to satisfy the following conditions,

17 $${M}_{{\rm{e}}}(\varphi )={M}_{{\rm{m}}}^{{\rm{p}}}(\varphi ,\omega ),$$

18 $${\eta }_{{\rm{M}}}^{{\rm{e}}}={\eta }_{{\rm{m}}}^{{\rm{p}}}(\varphi ,\omega ),$$

with *ρ*_m_ = *ρ*_e_. It is worth noting that, *ρ*_e_ is the function of *ϕ*, the required ${M}_{{\rm{m}}}^{{\rm{p}}}$ and ${\eta }_{{\rm{m}}}^{{\rm{p}}}$ are thus also the functions of *ϕ*.

Given the material parameters (*ρ*, *K* and *μ*) of the rubber matrix, metasurface thickness *l*_m_ and a specific angular frequency *ω*_p_, one can obtain the ideal *ϕ* by solving a polynomial equation derived by combining the equations (10), (15) and (17). Considering the multiple solutions of *ϕ*, only the solution satisfying 0 < *ϕ* < 1 should be chosen. Besides, a meaningful *ϕ* should also make sure the cavity radius *r*_c_ less than a half of the minimum of *l*_m_ and space period *d*. Then, using the solved *ϕ* and combining the equations (9), (16) and (18), the ideal *η* can further be acquired.

As an example, a rubber material is chosen with mass density *ρ* = 1100 kg/m^3^, Young’s modulus *E* = 30 MPa and Poison’s ratio *ν* = 0.497(These parameters correspond to bulk and shear modulus values of *K* ≅ 1.6667 GPa and *μ* ≅ 10.02 MPa); the material parameters of the steel plate are Young’s modulus *E*_s_ = 2.16 × 10^11^ Pa, mass density *ρ*_s_ = 7800 kg/m^3^ and Poisson’s ratio *ν*_s_ = 0.3; the thicknesses of rubber layer and steel plate are *l*_m_ = 30 mm and *l*_s_ = 30 mm; the density and sound speed of water are *ρ*_w_ = 1000 kg/m^3^ and *c*_w_ = 1489 m/s; the density and sound speed of air are *ρ*_*a*_ = 1.21 kg/m^3^ and *c*_*a*_ = 343 m/s. To achieve the perfect absorption at the specific frequency *f*_p_ = 500 Hz, the ideal parameters of the air-cavity based metasurface are solved as *ϕ*_p_ = 0.2255 and *η*_p_ = 0.5476. Based on these two parameters, the effective material parameters *ρ*_e_, *M*_e_, *μ*_e_ and *η*_e_ are 851.95 kg/m^3^, 53.298 MPa, 6.74 MPa and 0.5476, respectively. Prescribed the lattice constant *d* = 30 mm, the radius of the spherical air cavity can be deduced as *r*_c_ = 11.3 mm.

**Figure 2:**
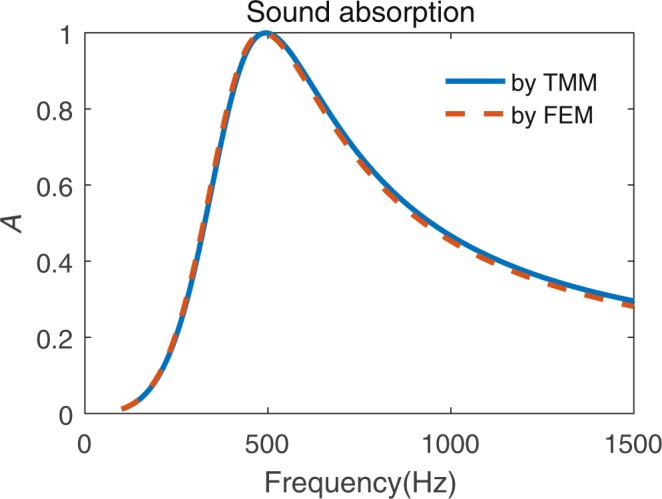
Sound absorption coefficients of the inverse designed cavity-based metasurface.

Figure 2 presents the sound absorption curves, calculated by a transfer matrix method (TMM) and the finite element method (FEM), of the inversely designed cavity-based metasurface. In the TMM, the effective parameters of the metasurface are acquired using a dynamic effective medium method (DEMM)^51^ to take the probable dynamic properties of the cavities into consideration. The results calculated by two methods coincide very well. A perfect absorption peak occurs at the prescribed frequency *f*_p_ = 500 Hz. This example verified the feasibility of the proposed inverse design approach. It is also interesting to note that, the metasurface thickness (0.03 m) accounts for only 1% of the working wavelength, revealing its deep subwavelength scale. Besides, although the metasurface is designed for a specific frequency, high absorption is manifested over a relatively broad frequency range. Over 80% of absorption can be achieved over the frequency range [394, 660] Hz, corresponding to a 53.2% relative bandwidth.

**Figure 3:**
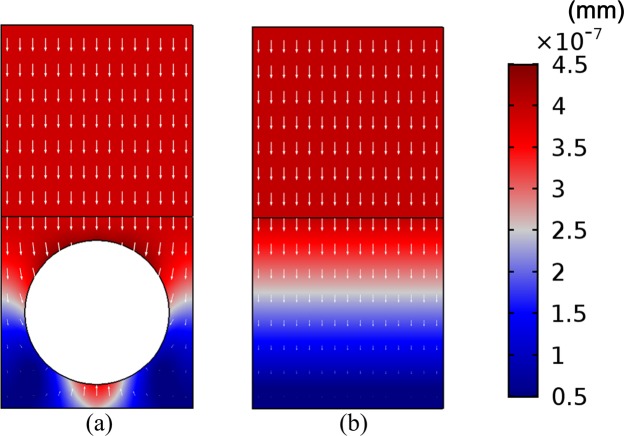
Displacement fields of a unit cell of the (**a**) cavity-based metasurface and (**b**) the equivalent homogeneous coating at 500 Hz.

Note that the resonance frequency of the embedded cavity can be predicted by formula^54^ ${f}_{{\rm{r}}}=\frac{{c}_{{\rm{t}}}}{\pi {r}_{{\rm{c}}}}=\frac{\sqrt{\mu /\rho }}{\pi {r}_{{\rm{c}}}}$ as *f*_r_ ≈ 2694 Hz, which is far away from the peak frequency in Fig. 2. Thus, the perfect absorption peak is not caused by the cavity resonance. Figure 3 further shows the displacement field on the yoz cross-section of a unit cell of the cavity-based metasurface at *f*_p_ = 500 Hz. The displacement field of the equivalent homogeneous layer of the cavity-based metasurface (with the material parameters equal to the effective ones of the cavity-based metasurface) is also plotted for comparison. Here the displatement fields are genereated by the FEM. The incident plane wave acts at the bottom of the structure, only the displacement fields in the solid domain are displayed. The linear color bar indicates the magnitude of the total displacement. The arrows, with length scaled to the displacement amplitude, denote the displacement vectors of the structure nodes at a certain time. It can be observed in Fig. 3(a) that, the steel plate vibrates along the z-axis as a rigid body with the equal amplitudes at every node, and the nodes at the bottom area (near the steel plate) of the metasurface show the larger amplitudes of displacements than the other parts. Figure 3(b) also shows the same pattern of vibration of the equivalent homogenous coating. Particularly, the equivalent homogenous layer is extended and compressed longitudinally due to the gradually varied displacement amplitudes. These phenomena provide an intuitive understanding of the vibration pattern of the lumped spring-mass oscillator model for the metasurface/steel plate system^46–48,55^, in which the mass is offered by the steel plate and the rubber metasurface plays the role of spring with proper damping. The embedded cavities are utilized to adjust the effective stiffness of the rubber matrix into the required one. As the resonances are usually accompanied by high energy density and improved impedance matching^56^, thus the perfect absorption can be attributed to the utilizing of the overall resonance mode of the metasurface/steel plate system.

**Figure 4:**
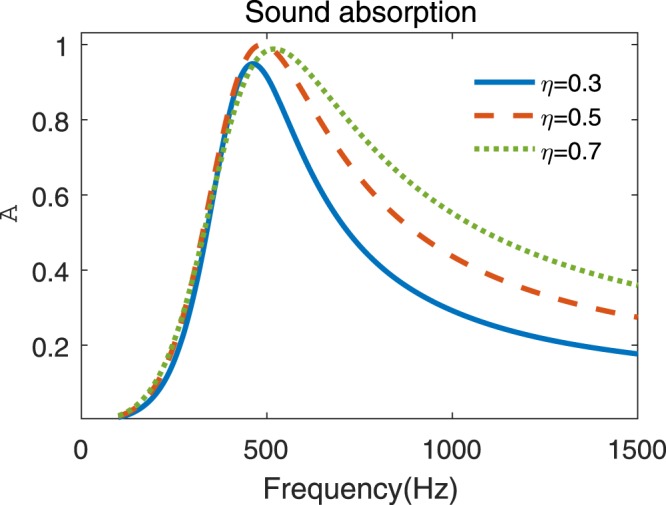
Sound absorption coefficients of the inverse designed metasurface with imperfect modulus loss factors.

It is also important to note that, it needs not only an ideal structure parameter, *ϕ*, but also an ideal material parameter, *η*, to obtain a perfect absorption at a specific frequency. However, it is also a usual situation that the loss factor of the given rubber material cannot meet the required one. Figure 4 shows the sound absorption curves of the inverse designed metasurface with various imperfect loss factors *η* = 0.3, 0.5, 0.7. As shown in the figure, three quasi-perfect absorption peaks occur around the specific frequency *f*_p_ = 500 Hz. This phenomenon illustrates that high absorption can still be achieved even if the loss factor of the given rubber material cannot meet the required one. Besides, the absorption coefficients at the comparatively higher frequencies also increase with *η*, leading to the broader relative bandwidths of over 80% of absorption, which are 34.2%, 51% and 58% for *η* = 0.3, 0.5, 0.7 respectively, although the peak frequency (*f*_peak_ = 462, 486, 520 Hz) of the absorption curve slightly increases with *η*. Thus, an overdamping rubber material (*η* > *η*_p_) is conductive to better absorption over a broad frequency range, although the totally perfect absorption cannot be achieved.

### Strategy for broadband perfect absorption and discussions

The above design can only realize perfect absorption at a specific frequency. The following investigations will show that a broadband perfect absorption can be realized utilizing the inherent frequency-dependent material parameters of viscoelastic rubber^57,58^. One can find from equations (15)~(18), with the given structural parameters *ϕ* and *l*_m_, the frequency-dependent *K* and *μ* are needed to satisfy the theoretical requirements for broadband perfect absorption. Replacing *K* and *μ* with the function of Young’s modulus *E* and Poison’s ratio *ν*, equation (15) can be rewritten as

19 $${M}_{{\rm{e}}}(\omega )=E(\omega )\cdot f(\varphi ,{l}_{{\rm{m}}},\nu ),$$

where *f*(*ϕ*, *l*_m_, *ν*) is a function unrelated to *E*. That’s to say, the effective longitudinal dynamic modulus *M*_e_(*ω*) is proportional to Young’s modulus *E*(*ω*) of rubber matrix. Combining equations (16), (18) and (19), two frequency-dependent requirements for material parameters of rubber matrix are demanded

20 $$E(\omega )=\frac{E({f}_{0})}{{M}_{{\rm{e}}}({f}_{0})}{M}_{{\rm{m}}}^{{\rm{p}}}({\varphi }_{{\rm{p}}},\omega ),$$

21 $$\eta (\omega )={\eta }_{{\rm{m}}}^{{\rm{p}}}({\varphi }_{{\rm{p}}},\omega ).$$

**Figure 5:**
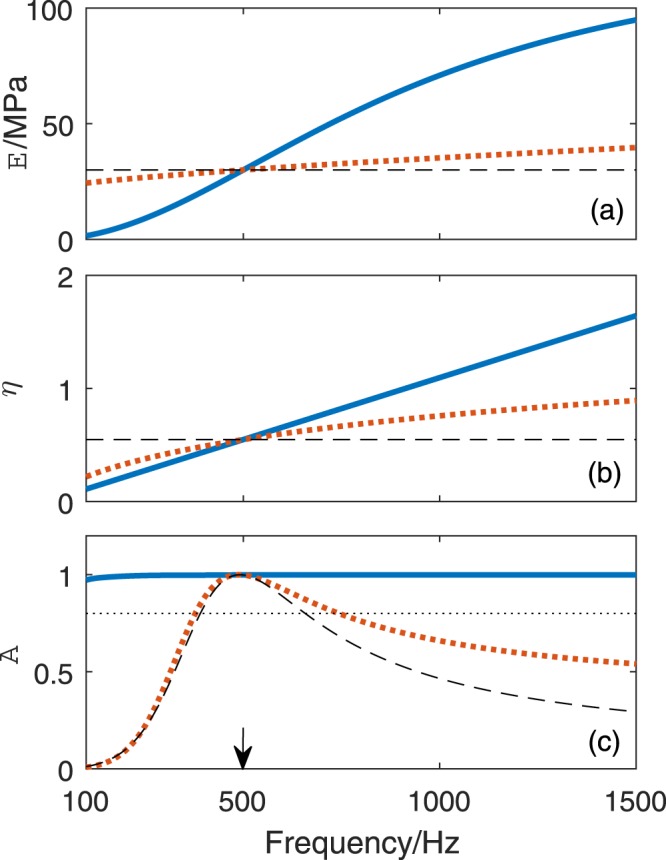
The frequency-dependent (**a**) dynamic modulus *E* and (**b**) loss factor *η* of the Young’s modulus of rubber matrix and (**c**) the corresponding sound absorption of the cavity-based metasurface (designed at *f*_p_ = 500 Hz). : Theoretical requirements for broadband perfect absorption, : frequency parameters prescribed by fractional Kelvin-Voigt model, : constant parameters. The black dotted horizontal line in (**c**) stands for the 80% of absorption. The black arrow points out the frequency position of perfect absorption.

Choose the designed case in Fig. 2 as an example, where the key structural parameters are *ϕ*_p_ = 0.2255 and *l*_m_ = 30 mm and the key material parameters are *M*_e_(*f*_p_) ≅ 53.298 MPa, *E*(*f*_p_) = 30 MPa and *η*_p_(*f*_p_) = 0.5476 at *f*_p_ = 500 Hz. Figure 5(a–c) show the frequency-dependent *E*, *η* and corresponding sound absorption coefficient *A* within the frequency band [100, 1500] Hz. It can be observed that both the dynamic modulus [*E*, 1.54–94.88 MPa] and loss factor [*η*, 0.1095–1.643] of Young’s modulus increase with frequency, correspondingly, the sound absorption coefficients of the metasurface with the prescribed frequency-dependent parameters are very close to unity above 100 Hz, implying a ultra-broadband perfect absorption.

Then, the problem becomes to design the rubber material to meet the required frequency-dependent parameters. It can be calculated that the slopes of the increase of ideal dynamic modulus-frequency curve and the increase of ideal loss factor-frequency curve plotted in a log-log coordinate system are close to 1.5 and 1, respectively. However, numerous experimental results showed that the slopes of dynamic mechanical parameters are generally smaller than unity^58–60^, and the most typical values of slope ranges from about 0.5 to 0.7^61^. Moreover, it is also been claimed that the dynamic modulus and loss properties of a rubber are interrelated through the so-called Kramers-Kronig relations^62,63^. Therefore, it is admittedly a challenging work to achieve the required parameters frequency by frequency. However, to the best of the authors’ knowledge, there is no fundamental barrier to develop a rubber material having the required frequency dependences of dynamic parameters. Since the conclusions that the larger the loss factor, the larger the frequency increase of dynamic modulus^58,62^, and in principle there are no bounds to increasing the loss factor maximum^62^. Thus, though very difficult, it is a meaningful challenge for the materials engineers to create such a kind of “meta-rubber”. Actually, this difficulty comes from the fact that, the embedding cavities used in this paper can only adjust the amplitudes of the effective parameters, but not the variation trends with frequency of a given rubber material within the concerned low-frequency range. If more complex embedded structures are used, such as the locally resonant units^51^, which can adjust the variation trends with frequency of the effective parameters of rubber layer, more degrees of freedom could be introduced into the design of the metasurface. The theoretical framework proposed in this paper with proper modifications can also be suitable for the rubber with other embedded structures.

The following statements will show that the broadband super-absorption can still be realized by properly designing the frequency position of perfect absorption of the cavity-based metasurface, when the frequency-dependent characteristics of the given rubber matrix cannot meet the required ones. A fractional Kelvin-Voigt model^63,64^, which is suitable to fit experimental curves in the low frequency range, is used to describe the dynamic behavior in the concerned frequency range. The frequency dependent dynamic modulus and loss factor prescribed by this model are^63,64^

22 $$E(\omega )={E}_{0}[1+\,\cos (\alpha {\rm{\pi }}/2){\omega }_{{\rm{n}}}^{\alpha }],$$

23 $$\eta (\omega )=\frac{\sin (\alpha {\rm{\pi }}/{\rm{2}}){\omega }_{{\rm{n}}}^{\alpha }}{1+\,\cos (\alpha {\rm{\pi }}/{\rm{2}}){\omega }_{{\rm{n}}}^{\alpha }},$$

where *ω*_n_ = *ωτ*_c_ is the normalized frequency with *τ*_c_ the creep time of rubber, *E*_0_ is the static modulus, and 0 < *α* < 1. This model has three independent parameters (*E*_0_, *α* and *τ*_c_). It can be seen that the slopes of frequency curves of dynamic properties are determined by *α*, and the larger the *α*, the larger the slopes. In this paper, a typical *α* = 0.7 is chosen^61^, and the frequency curves are prescribed to pass through the points (30 MPa, 500 Hz) and (0.5476, 500 Hz). Then, the static modulus and creep time of the rubber can be determined by Eqs (22) and (23) as *E*_0_ = 21.63 MPa and *τ*_c_ = 2.534 × 10^−4^ s. The new frequency dependent curves of *E* and *η* predicted by the fractional Kelvin-Voigt model are presented in Fig. 5(a,b), respectively. Smaller slopes of frequency dependences than the required ones can be observed. The corresponding absorption coefficients curve is also presented in Fig. 5(c). Over 80% of absorption can be achieved over the frequency range [378, 743] Hz corresponding to a 73% relative bandwidth, and the absorption coefficients in high frequencies are generally enhanced compared with those of the rubber with constant dynamic parameters. Thus, the inherent frequency-dependent parameters of rubber can be useful in broadening the bandwidth of high absorption, although the perfect absorption can only occur near 500 Hz.

**Figure 6:**
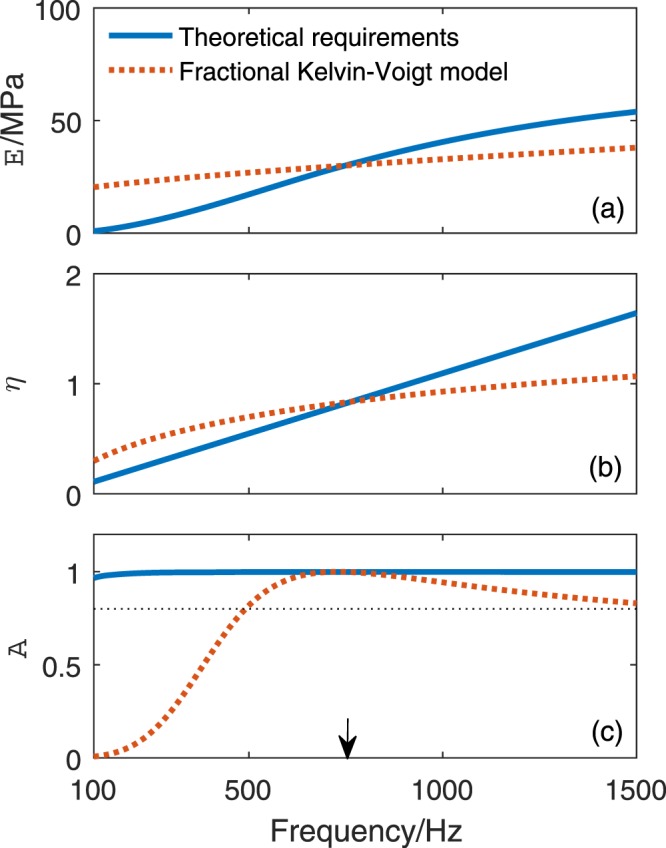
The frequency-dependent (**a**) dynamic modulus *E* and (**b**) loss factor *η* of the Young’s modulus of rubber matrix and (**c**) the corresponding sound absorption of the cavity-based metasurface (designed at *f*_*p*_ = 750 Hz). The black dotted horizontal line in (**c**) stands for the 80% of absorption. The black arrow points out the frequency position of perfect absorption.

In the second example, the frequency position of the perfect absorption is appointed at *f*_*p*_ = 750 Hz, while the reference parameters of the rubber matrix keep the same with those for the first example in Fig. 2. Based on the proposed inverse design method, the ideal parameters of the air-cavity based metasurface are solved as *ϕ*_p_ = 0.1338 and *η*_p_ = 0.831. Then, the ideal frequency dependent curves of dynamic modulus and loss factor for broadband perfect absorption are shown in Fig. 6(a,b). The required variation ranges of dynamic modulus and loss factor are respectively [*E*, 0.89–53.91 MPa] and loss factor [*η*, 0.1095–1.643]. As comparison, the frequency dependent curves prescribed by the fractional Kelvin-Voigt model, with a same *α* = 0.7, are also presented. It’s important to note that both groups of frequency curves are prescribed to pass through the points (30 MPa, 750 Hz) and (0.831, 750 Hz). Then, the static modulus and creep time of the rubber can be determined by Eqs (22) and (23) as *E*_0_ = 17.3 MPa and *τ*_c_ = 4.22 × 10^−4^ s. Compared with Fig. 5(a), the discrepancies between the frequency curve of theoretical required dynamic modulus and that prescribed by the fractional Kelvin-Voigt model become smaller as shown in Fig. 6(a). Similar situation can also be found for the loss factor by comparing Figs 5(b) and 6(b). Correspondingly, as shown in Fig. 6(c), over 80% of absorption can be achieved in the frequency range [490, 1500] Hz, corresponding to a 134.7% relative bandwidth. This example illustrates that the broadband super-absorption can still be realized by properly designing the frequency position of perfect absorption of the cavity-based metasurface, although the inherent frequency dependences of the dynamic properties of the rubber cannot meet the required ones for broadband perfect absorption.

## Conclusions

This paper focuses on the perfect absorption of low-frequency waterborne sound with ultrathin metasurface under a finite-thickness steel plate followed by semi-infinite air. Two theoretical requirements for broadband perfect absorption have been derived firstly. Based on the theoretical requirements, an acoustic metasurface, consisting of a rubber matrix embedded periodically with air cavities, is inversely designed to achieve perfect absorption at 500 Hz. The metasurface has a thickness of 1% of the working wavelength and maintains a substantially high absorptance over a relatively broad bandwidth. The perfect absorption peak is attributed to the overall resonance mode induced by the large inertia of the steel plate. High absorption can still be achieved even if the loss factor of the given rubber material cannot meet the ideal requirement. Furthermore, it is suggested that an ultra-broadband perfect absorption can be achieved by utilizing the inherent frequency-dependent characteristics of dynamic parameters of rubber matrix. If the frequency-dependent characteristics of the given rubber matrix cannot meet the required ones, a broadband super-absorption can still be realized by properly designing the frequency position of perfect absorption of the cavity-based metasurface.

## Methods

### Transfer matrix method

At first, a DEMM borrowed from ref.^51^ is used to obtain the more precise effective parameters (*ρ*_*e*_, ${\tilde{K}}_{e}$ and ${\tilde{\mu }}_{e}$) of the metasurface. In this method, the air medium in the inner cavities and the corresponding acoustic-structure boundary conditions are considered. Then, a simple one-dimensional transfer matrix approach is used to calculate the acoustic performances under normal incidence. The transfer matrix of the (n−1)th layer, **T**_*n*−1_, relates the output pressure and velocity to the input pressure and velocity by^50,53,65^

24 $$\{\begin{array}{c}{p}_{n-1}\\ {v}_{n-1}\end{array}\}={{\bf{T}}}_{n-1}\{\begin{array}{c}{p}_{n}\\ {v}_{n}\end{array}\}=[\begin{array}{cc}\cos ({k}_{n-1}^{\ast }{l}_{n-1}) & {\rm{j}}{Z}_{n-1}\,\sin ({k}_{n-1}^{\ast }{l}_{n-1})\\ \frac{{\rm{j}}\,\sin ({k}_{n-1}^{\ast }{l}_{n-1})}{{Z}_{n-1}} & \cos ({k}_{n-1}^{\ast }{l}_{n-1})\end{array}]\{\begin{array}{c}{p}_{n}\\ {v}_{n}\end{array}\}$$

where ${k}_{n-1}^{\ast }$, *l*_*n*−1_ and ${Z}_{n-1}={\rho }_{n-1}{c}_{n-1}^{\ast }$ are respectively the wave number, thickness and impedance of (n−1)th layer, with *ρ*_*n*−1_ and ${c}_{n-1}^{\ast }$ being the density and longitudinal wave speed of this layer. The full transfer matrix **T** for the multi-layer structure is obtained as the product of the individual transfer matrix for the layers. With the assumption of an anechoic termination of the outgoing waves, the transmission and reflection coefficients for the periodically voided material modeled as a layer composite are, respectively, obtained as^50^

25 $$\tilde{T}=2{({t}_{11}+\frac{{t}_{12}}{{Z}_{a}}+{Z}_{w}{t}_{21}+\frac{{Z}_{w}}{{Z}_{a}}{t}_{22})}^{-1}$$

26 $$\tilde{R}=\frac{{t}_{11}+\frac{{t}_{12}}{{Z}_{a}}-{Z}_{w}{t}_{21}-\frac{{Z}_{w}}{{Z}_{a}}{t}_{22}}{{t}_{11}+\frac{{t}_{12}}{{Z}_{a}}+{Z}_{w}{t}_{21}+\frac{{Z}_{w}}{{Z}_{a}}{t}_{22}}$$

where *t*_11_, *t*_12_, *t*_21_ and *t*_22_ are the four elements of the full transfer matrix **T** and *Z*_*w*_, *Z*_*a*_ are the impedances of water and air, respectively. The sound absorption coefficient *A* can then be calculated from

27 $$A=1-{|\tilde{R}|}^{2}-\frac{{{\rm{Z}}}_{w}}{{Z}_{a}}{|\tilde{T}|}^{2}$$

### Finite element method

When the unit cell for periodic expansion is determined, the finite element software COMSOL Multiphysics® (v5.1)^66^ is used to model this problem. The rubber matrix is modeled as a solid domain, the semi-infinite water and air domains are fluid media. To be more rigorous, the inner cavities are also modeled as fluid media. The fully coupled acoustic-structure boundary conditions are applied at the interfaces of the solid and fluid domains. Only finite domains of water and air are modeled with the perfectly matched layers added at both the top of water domain and the bottom of air domain to mimic anechoic termination of outgoing waves^50,67^. Periodic boundary conditions are applied on the boundaries of the unit cell in corresponding directions. Solving the problem, one can obtain the displacement **u**_**e**_ for the structure and the pressure **p**_**e**_ in the fluid, and the reflection $\tilde{R}$ and transmission coefficients $\tilde{T}$ can also be obtained by measuring the reflected and transmitted pressure on the corresponding boundary^50^. The sound absorption coefficient *A* can then be calculated from equation (27).

## Data Availability

The data that support the findings of this study are available from the corresponding author upon reasonable request.
